# Whole-genome expression analyses of type 2 diabetes in human skin reveal altered immune function and burden of infection

**DOI:** 10.18632/oncotarget.16118

**Published:** 2017-03-11

**Authors:** Chun Wu, Xiaopan Chen, Jing Shu, Chun-Ting Lee

**Affiliations:** ^1^ Department of Molecular and Cellular Pharmacology, Miller School of Medicine, University of Miami, Miami, FL, USA; ^2^ Department of Reproductive Endocrinology, Zhejiang Provincial People's Hospital, Hangzhou Medical College, Hangzhou, P.R. China; ^3^ Department of Neurology, Miller School of Medicine, University of Miami, Miami, FL, USA

**Keywords:** transcriptome, type 2 diabetes, human skin, immune, infection

## Abstract

Skin disorders are among most common complications associated with type 2 diabetes mellitus (T2DM). Although T2DM patients are known to have increased risk of infections and other T2DM-related skin disorders, their molecular mechanisms are largely unknown. This study aims to identify dysregulated genes and gene networks that are associated with T2DM in human skin. We compared the expression profiles of 56,318 transcribed genes on 74 T2DM cases and 148 gender- age-, and race-matched non-diabetes controls from the Genotype-Tissue Expression (GTEx) database. RNA-Sequencing data indicates that diabetic skin is characterized by increased expression of genes that are related to immune responses (*CCL20*, *CXCL9*, *CXCL10*, *CXCL11*, *CXCL13*, and *CCL18*), JAK/STAT signaling pathway (*JAK3*, *STAT1*, and *STAT2*), tumor necrosis factor superfamily (*TNFSF10* and *TNFSF15*), and infectious disease pathways (*OAS1*, *OAS2*, *OAS3*, and *IFIH1*). Genes in cell adhesion molecules pathway (*NCAM1* and *L1CAM*) and collagen family (*PCOLCE2* and *COL9A3*) are downregulated, suggesting structural changes in the skin of T2DM. For the first time, to the best of our knowledge, this pioneer analytic study reports comprehensive unbiased gene expression changes and dysregulated pathways in the non-diseased skin of T2DM patients. This comprehensive understanding derived from whole-genome expression profiles could advance our knowledge in determining molecular targets for the prevention and treatment of T2DM-associated skin disorders.

## INTRODUCTION

Type 2 diabetes mellitus (T2DM) accounts for ˜ 90% of diabetes and constitutes a great challenge for healthcare systems. T2DM is a principle contributor to heart disease, neuropathy, eye failure, and nephropathy [1]. Skin infections and other diabetes-associated skin disorders are among the most common complications, affecting 30 to 70% of people with diabetes [2]. Certain skin disorders are clearly more frequent in diabetic patients, but little is known about the molecular mechanisms underlying this association [3]. Due to the impaired wound healing in diabetic skin, chronic ulcers are one of the most severe cutaneous lesions faced by people with T2DM [4]. Hence, a better understanding of the mechanisms underlying the greater susceptibility of T2DM patients to developing skin disorders should lead to a better management or prevention of such complications.

There is a consensus that the mechanical competence of the dermis is reduced in diabetes [3]. A study led by Bermudez et al. found that db/db diabetic mice skin is biomechanically inferior to nondiabetic skin [5]. It has been suggested that altered collagen expression may contribute to this reduced mechanical competence; however, results of the expression of dermal collagen are not consistent [5, 6]. Furthermore, a recent study showed an increased number of skin inflammatory cells in patients with type 1 or type 2 diabetes, indicating a more permissive barrier in DM [7].

In this study, we hypothesized that T2DM is associated with dysregulated gene networks, which underlie the higher risk of developing cutaneous diseases and skin infections. With the rapid development of high-throughput sequencing technologies and precision medicine, scientists are now increasingly able to understand genetic and environmental factors that may contribute to complex diseases. We performed differential expression and pathway enrichment analyses on 74 T2DM cases and 148 non-DM controls from the Genotype-Tissue Expression (GTEx) database [8]. The GTEx project provides a wealth of gene expression and associated extensive clinical data [9]. We have previously performed extensive bioinformatics analyses using the GTEx database and successfully demonstrated that type 2 diabetic skeletal muscle is characterized by insulin resistance [10], which is considered to be the primary defect involved in T2DM [11]. To the best of our knowledge, our analysis is the first, and currently the largest, RNA-Seq-based transcriptome study in the skin of T2DM patients. RNA-Seq results provided 182 significant differentially expressed (DE) genes in the skin of T2DM. These dysregulated gene expression profiles could help in determining molecular targets for the prevention and the development of potential therapeutic avenues of T2DM-associated skin disorders.

## RESULTS

### Differential expression analysis identified 182 significant DE genes in the human skin of T2DM

**Figure 1:**
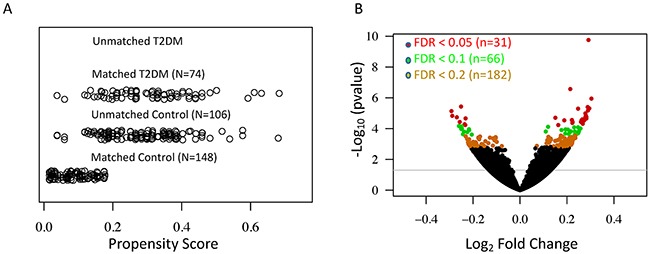
Differential expression analyses reveal a large number of significant T2DM-associated genes in the human skin **(A)** Jitter plot shows the distribution of propensity scores in the T2DM and control groups. **(B)** Volcano plot shows the –log10 (*P-*value) and log_2_ fold change for all expressed genes. Differentially expressed genes in the T2DM at the FDR level of 0.05, 0.1, and 0.2 are indicated by red, green and yellow respectively.

We examined expression changes of 56,318 transcribed genes (on the basis of Gencode V19 annotation, TrueSeq V1) in skin samples (lower leg, N=357) from the GTEx RNA-Seq database. To reduce the bias due to covariates in the generalized linear regression model, we used an optimal matching algorithm to balance gender, race, and age between T2DM and control groups (Supplementary Table 1), which minimized the average of distances among matched units [12]. The jitter plot in Figure 1A shows a similar overall distribution of propensity scores in matched control (N=148) and T2DM (N=74). After removing unmatched controls (N=106), we performed generalized linear regression of normalized read counts for each expressed gene against disease status, adjusting for known and hidden surrogate variables [13]. The volcano plot in Figure 1B shows significant T2DM-related gene expression changes in the skin. At a stringent FDR level of 0.2, we identified 182 significant DE genes in the type 2 diabetic skin (Supplementary Table 2).

### Validation by microarray dataset

**Figure 2:**
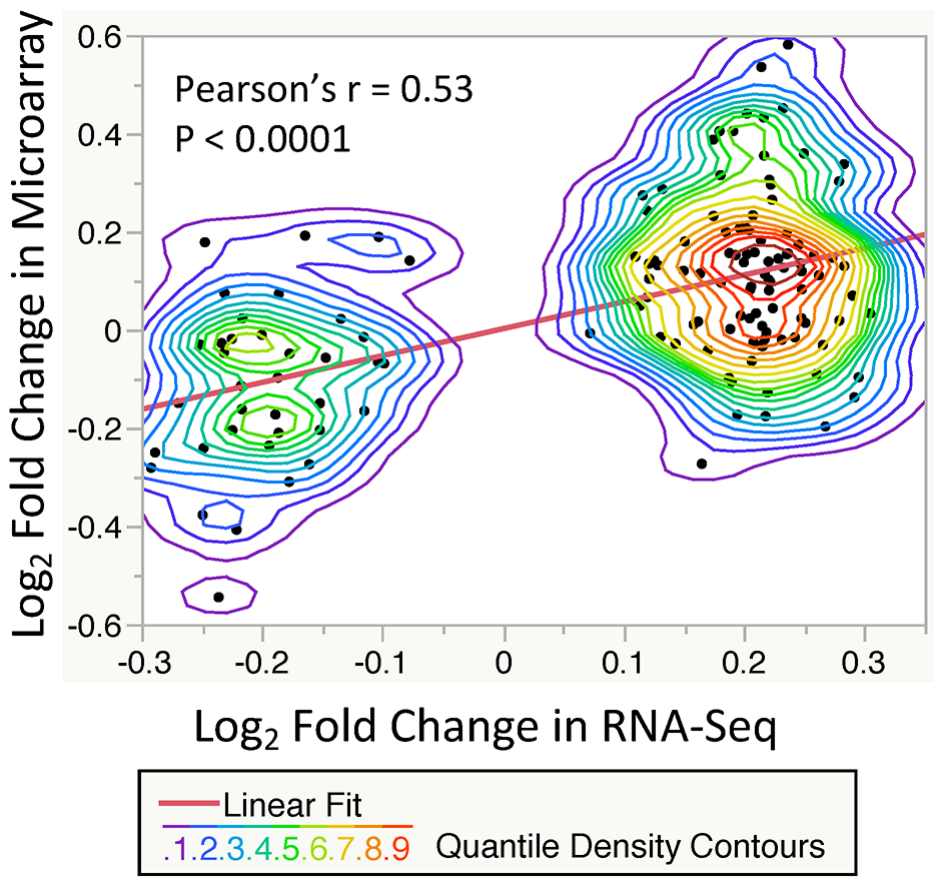
Correlation of significant T2DM-related gene expression changes (FDR < 0.2, n = 135) between the RNA-Seq and Microarray datasets Each point showing the log_2_ fold change between T2DM and control subjects. A significant correlation is observed with *P-*value < 0.0001 and Pearson's *r* = 0.53 (Linear fit line slope = 0.55). The nonparametric density contour lines are quantile contours in 5% intervals.

To validate our results of significant T2DM-related gene expression changes, we compared our findings with the microarray-based gene expression GTEx dataset. The GTEx study has demonstrated a strong correlation (Pearson's r = 0.83) between RNA-Seq and Microarray platforms [8]. We pulled out 58 skin samples (lower leg) with microarray-based transcriptome profiles, then applied the same exclusion criteria and preprocesses, resulting in 12 T2DM cases and 24 Non-DM controls. We performed a similar linear regression-based approach to model gene expression values against T2DM status. As shown in Figure 2, Log_2_ Fold Changes (LFCs) of our DE genes were significantly correlated with LFCs of common genes (n=135) in the microarray dataset (Pearson's Correlation = 0.53). These results suggest a consistency of T2DM-related gene expression changes between these two platforms.

### Pathway enrichment analysis revealed altered immune function and pathways related to infectious diseases in T2DM

**Figure 3:**
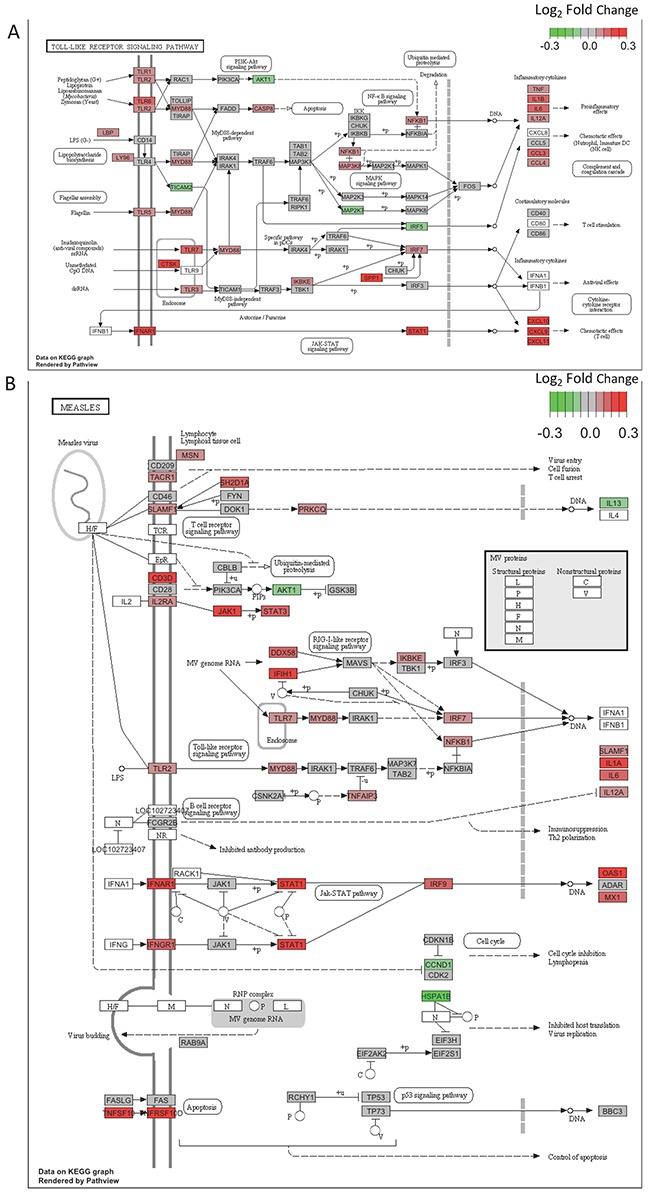
**(A)** DE genes are significantly overrepresented in the Toll-like receptor signaling pathway. Pathview is used to visualize gene expression changes. Color key indicates log_2_ fold change. Up-regulated genes appear in red, and down-regulated genes appear in green. **(B)** Genes in the Measles pathway show a concordant up-regulation in T2DM. Pathview is used to visualize gene expression changes. Color key indicates log_2_ fold change. Up-regulated genes appear in red, and down-regulated genes appear in green.

To obtain a functional overview of these significant T2DM-associated DE genes (FDR<0.2), we conducted overrepresentation tests on up- and down-regulated genes separately. As shown in Table 1, up-regulated genes were significantly overrepresented in two categories: the immune response and infectious disease pathways (*q*-value < 0.05). Genes in two significant T2DM-associated KEGG pathways, Toll-like receptor signaling pathway (hsa04620, Figure 3A) and Measles pathway (hsa05162, Figure 3B), were visualized through “pathview” R package [14]. To determine whether the FDR cutoff affects the pathway enrichment analysis, we performed overrepresentation test on 66 DE genes with FDR < 0.1. As shown in Supplementary Table 3, up-regulated DE genes were significantly enriched in the similar immune response and infectious diseases pathways as observed with FDR < 0.2. These results indicate an activation of infectious disease-related immune functions in type 2 diabetic skin. Moreover, gene set enrichment analysis, an approach which determines whether a group of genes has significant concordant differences, showed coordinated up-regulation of genes in all of these immune response and infectious disease pathways (Supplementary Table 4).

**Table 1:** Significantly enriched pathways on upregulated DE genes (FDR < 0.2) in T2DM

ID	Description	*P*-value	*q*-value	Gene ID	Count
**Immune System**					
hsa04062	Chemokine signaling pathway	1.15E-07	1.40E-05	STAT1/CCL20/CXCL9/CXCL10/CXCL11/CXCL13/ADCY1/LYN/STAT2/CCL18/JAK3	11
hsa04620	Toll-like receptor signaling pathway	1.41E-04	4.26E-03	CTSK/STAT1/CXCL9/CXCL10/CXCL11/SPP1	6
hsa04060	Cytokine-cytokine receptor interaction	8.06E-04	1.63E-02	CCL20/TNFSF10/CXCL9/CXCL10/CXCL11/CXCL13/TNFSF15/CCL18	8
**Infection**					
hsa05164	Influenza A	5.98E-06	3.00E-04	RSAD2/IFIH1/STAT1/TNFSF10/CXCL10/STAT2/OAS1/OAS3/OAS2	9
hsa05162	Measles	7.44E-06	3.00E-04	IFIH1/STAT1/TNFSF10/STAT2/OAS1/OAS3/OAS2/JAK3	8
hsa05168	Herpes simplex infection	4.80E-04	1.16E-02	IFIH1/STAT1/SP100/STAT2/OAS1/OAS3/OAS2	7

Although there were no enriched pathways among down-regulated genes, cell adhesion molecules (CAMs) pathway was the most significant pathway with *P*-value = 0.069 (*q*-value = 0.417). The expression of cell adhesion molecules, *NCAM1* (neural cell adhesion molecule 1) and *L1CAM* (L1 cell adhesion molecule), were significantly decreased in T2DM (Supplementary Table 2). Moreover, the expression of the collagen-related genes including *PCOLCE2* (procollagen C-endopeptidase enhancer 2), *COL9A3* (collagen type IX alpha 3), and *COL22A1* (collagen type XXII alpha 1) were significantly changed in T2DM (Supplementary Table 2). These results indicate altered lymphocyte trafficking and structural changes in dermal collagen in the skin of T2DM.

## DISCUSSION

Our study shows for the first time T2DM-related whole-genome gene expression changes in the human skin. Although it is well known that patients with T2DM are at particular risk of developing skin infections and disorders, the molecular mechanisms leading to this complication are still poorly understood. The clearest trend in our RNA-Seq analysis is the significant enrichment of up-regulated genes in the immune response and infectious disease pathways.

T2DM is associated with increased systemic inflammation, which has been suggested to induce insulin resistance [15], the key feature of T2DM. Although a recent study indicates that increased inflammation was found in the skin of patients with T2DM [7], the molecular insights into the inflamed skin of T2DM remain unclear. Here we showed that three KEGG immune-related pathways including chemokine signaling pathway (hsa04062), toll-like receptor signaling pathway (hsa04620), and cytokine-cytokine receptor interaction (hsa04060) were significantly up-regulated in the skin of T2DM patients (Table 1). Notably, among significantly up-regulated DE genes in immune response pathways, chemokines including *CCL20, CXCL9, CXCL10, CXCL11, CXCL13*, and *CCL18* (Table 1 and Supplementary Table 2) have been suggested to induce leukocyte chemotaxis and activation [16]. Specifically, chemokines *CXCL9*, *CXCL10*, and *CXCL11* were involved in the T cell recruitment and infiltration in inflammatory skin diseases [17]. Our expression profiles indicated that JAK/STAT molecules including *JAK3*, *STAT1*, and *STAT2* were up-regulated in the skin of T2DM (Table 1 and Supplementary Table 2). The JAK/STAT signaling pathway is associated with cellular functions such as proliferation, differentiation, and migration [18]. Importantly, the JAK-STAT pathway has been shown to dysregulate the immune response in the chronic inflammatory skin disorders [19]. Moreover, tumor necrosis factor superfamily members including *TNFSF10* and *TNFSF15*, which play essential roles in stimulating T and B lymphocytes [20], were up-regulated in T2DM (Table 1 and Supplementary Table 2). Increased expression of chemokines, JAK/STAT molecules, and TNFSF genes indicates inflammatory dysregulation of leukocytes in the skin of T2DM. Altered expression of immune-related genes may underlie the greater susceptibility of T2DM for developing infectious diseases.

A number of studies have shown that patients with T2DM are associated with a higher risk of infectious diseases [21–24]. About fifty percent of the diabetic patients involved in Shah's study had at least one hospitalization or physician claim for infection in each cohort year [23]. Another prospective cohort study in Australia showed that the risk of hospitalization for an infectious disease increased more than two-fold in diabetic patients [22]. Furthermore, infection-related mortality has been shown to be higher in patients with diabetes [24]. Although the hyperglycemic environment is suggested to cause immune dysfunction that increases the risk for diabetic patients acquiring infectious diseases, the molecular mechanisms underlying the relationship between glycaemia and infections are still poorly understood [25]. We identified three infectious disease pathways that were significantly enriched among up-regulated DE genes (Table 1). Notably, three genes, *OAS1, OAS2*, and *OAS3* of the 2'-5'-oligoadenylate synthetase family, were significantly up-regulated in T2DM and presented in all of these infectious disease pathways (Table 1 and Supplementary Table 2). The 2'-5'-oligoadenylate synthetase and its downstream effector enzyme RNase L are known to be involved in the host defense mechanisms against viral infection [26]. The activity of 2'-5'-oligoadenylate synthetase was found to be persistently activated in type 1 diabetes [27]. Moreover, another antiviral gene, *IFIH1* (interferon induced with helicase C domain 1), was significantly up-regulated in patients with T2DM and involved in all infectious disease pathways (Table 1 and Supplementary Table 2). *IFIH1* is a member of the RIG-I-like receptor (RLR) family involved in the recognition of viral RNA and mediates the virus-induced innate immune response [28]. It has been shown that rare variant or reduced expression of *IFIH1* protects against type 1 diabetes [29, 30]. However, the influences of these antiviral genes in T2DM are not clear. Excessive expression of OAS family genes and *IFIH1* may limit the magnitude of the response to viruses in the skin of T2DM, leading to a higher risk of viral infection.

Importantly, we observed the similar immune activation in response to infections in the human skeletal muscle of T2DM [10]. Three infectious disease pathways, Influenza A (hsa05164), Measles (hsa05162) and Herpes simplex infection (hsa05168), were significantly up-regulated in both muscle and skin of type 2 diabetic patients. In addition, 152 DE genes in the skin (FDR < 0.2), which are also expressed in the muscle, showed a consistent T2DM-associated expression changes between the muscle and skin (Supplementary Figure 1). These results indicated the burden of infection in both human skin and muscle of T2DM, and are consistent with high rates of skin and soft-tissue infections observed in patients with type 2 diabetes [21].

Although no pathways were significantly enriched among down-regulated genes, Cell Adhesion Molecules (CAMs) pathway was placed at the top of all down-regulated pathways from overrepresentation tests (*q*-value = 0.417). CAMs are cell surface proteins involved in mediating leukocyte migration in the immune system [31]. Notably, the expression of *NCAM1* was significantly down-regulated in the skin of T2DM (Supplementary Table 2). NCAM1 is expressed in NK cells and serves as a host protective component in the local innate immune response against viral infections [32]. Moreover, the expression of another cell adhesion molecule *L1CAM* was significantly down-regulated in T2DM (Supplementary Table 2). L1CAM is expressed on the surface of leukocytes and regulates the adhesion of leukocytes through L1 and L1/NCAM homophilic binding [33]. Decreased expression of cell adhesion molecules in T2DM may underlie a greater susceptibility to the development of infectious diseases. In addition, two collagen-related genes, *PCOLCE2* and *COL9A3* were significantly down-regulated in T2DM, while *COL22A1* was significantly up-regulated (Supplementary Table 2). Altered expression of collagen-related genes may affect the physical integrity of the epidermis, leading to greater susceptibility of T2DM patients to infectious diseases.

In conclusion, our RNA-Seq analyses of the human skin from 74 T2DM cases and 148 matched controls revealed comprehensive molecular and network defects associated with T2DM. We acknowledge that gene expression studies in the human postmortem tissues alone do not allow establishing the causal relationship with the type 2 diabetes [33]. Nerveless, skin and soft tissue infections are prominent in T2DM patients with chronic hyperglycemia, and hyperglycemia has been shown to affect immune responses and increase susceptibility to infections in patients with T2DM [34]. Therefore, it is reasonable for us to hypothesize that the up-regulation of genes related to immune response and infectious disease pathways partly reflect the adaptive changes caused by the type 2 diabetic states such as hyperglycemia. Skin, as the most visible and largest organ of the integumentary system, can be considered as the first warning signal for T2DM and a good predictor marker for evaluating the therapy efficiency in patients with T2DM. This analytical study provides novel molecular targets for developing therapeutics in the prevention and management of T2DM-associated skin disorders. In addition, given the easy accessibility of skin, genes whose expression levels significantly changed in T2DM can be employed as potential diagnostic biomarkers for T2DM.

## MATERIALS AND METHODS

### GTEx database

The GTEx database (v6, October 2015 release) contains 357 skin samples from lower leg with RNA-Seq transcriptome profiles. Detailed information on sample collection, RNA sequencing, and the data processing pipeline can be found in the GTEx Consortium paper [8]. We excluded cases with type 1 diabetes, unknown T2DM status, and races other than black or white, leaving 254 non-diabetic samples and 74 T2DM cases. To reduce effects of cofounders in our statistical model, MatchIt (v2.4) in R was used to balance three covariates (age, gender, and race) between Non-DM controls and T2DM cases with “optimal” matching and 2:1 optimal ratio (Supplementary Table 1).

### Identification of significantly differentially expressed genes in T2DM

Differentially expressed genes were identified as described previously [10]. Briefly, we used the “svaseq” function from the sva R package to identify hidden cofounding factors [13]. In addition to gender, 5 surrogate variables were added to the formula in DESeq2 [35]. The residuals for normalized read counts, after gender and surrogate variables correction, were tested against the “T2DM” status using the following negative binomial (NB) generalized linear regression model (GLM):

$$K_{\mathrm{ij}}\sim \mathrm{NB}\left(\mu_{\mathrm{ij}}{,\alpha }_{j}\right)$$ (1)

$$\mu_{ij}=s_{i}q_{ij}$$ (2)

$$\log_{2}\left(q_{\mathrm{ij}}\right){=\beta }_{0}{}_{j}{+\beta }_{1j}{\text{T2DM}}_{i}{+\beta }_{2}{}_{j}{\text{GENDER}}_{i}+\sum_{k}^{5}1\gamma_{\mathrm{kj}}{\mathrm{SV}}_{\mathrm{ki}}+\varepsilon_{\mathrm{ij}}$$ (3)

Where K_ij_ is the read count for gene j in sample i, fitted with a negative binomial distribution. α_j_ is a gene-specific dispersion parameter. μ_ij_ represents fitted mean, containing a sample-specific size factor s_i_ and a covariate-dependent part q_ij_ [35]. In Equation 3, β_0_ is the regression intercept for gene j, ε_ij_ is the error term. β_1j_, β_2j_, and γ_kj_ (k=1, …, 5) denote the regression coefficients of T2DM, gender, and k_th_ surrogate variables for gene j respectively. The Wald test *P-*values were adjusted for multiple testing using the Benjamini-Hochberg (BH) algorithm. We defined significant DE genes at the level of FDR < 0.2.

To validate our results, we extracted the microarray-based GTEx dataset from the skin of lower leg, which contains 12 subjects with T2DM and 46 Non-DM controls [36]. MatchIt (v2.4) in R was used to balance three covariates (age, gender, and race) with “optimal” matching and 2:1 optimal ratio, resulting 12 T2DM and 24 Non-DM controls. For each gene signature, we performed differential expression analysis by using the limma R package [37]. Multiple testing *P-*values were adjusted using the BH method.

### Detecting significantly enriched KEGG pathways in T2DM

We performed overrepresentation tests on significant DE genes (FDR < 0.2) in T2DM by using clusterProfiler (v3.0.2) in R [38]. The function “enrichKEGG” was used to test whether up- (n=122) or down-regulated (n=60) DE genes are significantly overrepresented in given pathways from Kyoto Encyclopedia of Genes and Genomes (KEGG PATHWAY database). *q*-values were reported for FDR control. For pathway-based analyses, we defined significant T2DM-associated biological functions at the level of *q*-value less than 0.05. Selected significant T2DM-associated pathways were visualized through the “pathview” R package [14].

Gene set enrichment analysis (GSEA) was performed by using *clusterProfiler* (v3.0.2) in R [38], which implements the algorithm developed by the Broad Institute [39]. Specifically, we constructed a pre-ranked gene list of all expressed genes ordered by log_2_ fold change from DESeq2 package. Enrichment score (ES) and significance level of ES (nominal P value) were calculated by 1000 phenotype-based permutation test. Pre-defined pathways from KEGG PATHWAY database with minimal gene size of 10 and maximal of 500 was used in GSEA. *q*-values were calculated for FDR control. Significant pathways with *q*-value less than 0.05 were reported (Supplementary Table 3).

### Statistical analysis and dataset access

All statistical computing was performed in the R (v3.3, https://www.r-project.org/) and JMP (v12, SAS Institute). The GTEx dataset can be downloaded from dbGaP, study accession no. phs000424.v6.p1.

## SUPPLEMENTARY MATERIALS FIGURES AND TABLES










