# Supplementary material for: Whole-genome expression analyses of type 2 diabetes in human skin reveal altered immune function and burden of infection
Source: Oncotarget. 2017 Mar 11;8(21):34601–9. doi: 10.18632/oncotarget.16118 (PMC5470994; doi:10.18632/oncotarget.16118)
Supplement: Supplementary file 1 [file oncotarget-08-34601-s001.pdf]

Whole-genome expression analyses of type 2 diabetes in human skin reveal altered immune function and burden of infection

SUPPLEMENTARY FIGURE AND TABLES

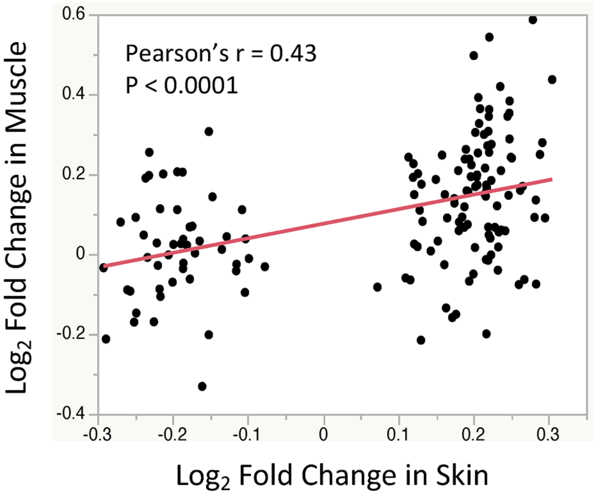

Supplementary Figure 1: Correlation of significant T2DM-related gene expression changes between the skin and muscle datasets on skin DE genes (FDR < 0.2, n = 152). Each point showing the $\log_2$ fold change between T2DM and control subjects. A significant correlation is observed with *P*-value < 0.0001 and Pearson's *r* = 0.43 (Linear fit line slope = 0.36).

Supplementary Table 1: Demographic characteristics of matched subjects.

See Supplementary File 1

Supplementary Table 2: Significant DE genes (padj < 0.2) associated in T2DM.

See Supplementary File 2

Supplementary Table 3: Significantly enriched pathways on upregulated DE genes (padj < 0.1) in T2DM.

See Supplementary File 3

Supplementary Table 4: Gene set enrichment analysis (q-value < 0.05).

See Supplementary File 4
